# HONORING OUR HEALTH AND HISTORY: FRAMING BRAIN HEALTH THROUGH A CULTURALLY CELEBRATORY LENS

**DOI:** 10.1093/geroni/igae098.1266

**Published:** 2024-12-31

**Authors:** Raina Croff, Patrice Fuller, Charles Fennell, Taryn Gordon

**Affiliations:** Oregon Alzheimer’s Disease Research Center, Oregon Health & Science University, Portland, Oregon, United States; Oregon Health & Science University, Portland, Oregon, United States; Oregon Health & Science University, Portland, Oregon, United States; George Fox University, Newberg, Oregon, United States

## Abstract

Asset-based, culturally celebratory frameworks center community strengths, history, culture, and community resilience. Applying such frameworks to Black American brain health engenders agency over individual and community health, and empowers older Black adults to engage in aging research. Amidst gentrification, such frameworks become especially salient. The Sharing History through Active Reminiscence and Photo-imagery (SHARP) Study uses Black history to engage older Black adults in walking social reminiscence for brain health within gentrifying neighborhoods in Portland and Seattle. Since 2016, SHARP has engaged 82 healthy and cognitively impaired walkers, including dementia family caregivers. Triads walked 1-mile routes 3x/week for 1, 4, or 6 months using the SHARP walking application on a group tablet. GPS-linked images of local Black history and culture prompted social reminiscence during walking. Walking narratives were recorded for an oral history archive. This presentation explores SHARP’s culturally celebratory framework informed by Black Lives Matter principles applied to health, Asset-Based Community Development, and Africana Worldview. We examine how centering Black history over Black health deficits, community memory over individual memory loss, and group participation over individual research participation produced high retention, re-enrollment rates, and recommendations. We apply theoretical concepts and study design to understand participant experiences, including how impromptu neighborhood encounters during walks mimicked social patterns from younger years, recalling joyful community memories despite gentrification, improved coping with change, and decreased psychological burden of exercise. Finally, we highlight challenges to the SHARP approach, including balancing a culturally celebratory framework with duly addressing the heavy burden of Alzheimer’s and gentrification.

